# Factors associated with post-stroke depression one month after discharge among older patients based on Health Ecology Theory: a retrospective cross-sectional study of consecutive cases

**DOI:** 10.3389/fpsyt.2026.1919008

**Published:** 2026-08-06

**Authors:** Yunlei Zhang, Yao Liao, Huanhuan Liu

**Affiliations:** 1 Department of Neurology, Jingzhou Central Hospital, Jingzhou, China; 2 Department of Cardiothoracic and Vascular Surgery, Jingzhou Central Hospital, Jingzhou, China

**Keywords:** consecutive cases, cross-sectional study, health ecology theory, older patients, post-stroke depression

## Abstract

**Objective:**

This study aimed to examine multidimensional factors associated with post-stroke depression one month after discharge among older patients within the framework of Health Ecology Theory.

**Methods:**

This retrospective cross-sectional study consecutively included 482 older stroke patients (aged ≥ 65 years) who were hospitalized at a tertiary hospital in Jingzhou, China, between January and December 2025. The Patient Health Questionnaire-9 (PHQ-9) was used to evaluate the level of depressive symptoms at one month after discharge, with a score of ≥10 used as the study-defined criterion for PSD. Potential factors were systematically categorized into five dimensions according to Health Ecology Theory: personal characteristics (sex, age, body mass index [BMI], comorbidities, acute treatment, functional disability via the modified Rankin Scale [mRS], and early cognitive impairment assessed using the Eight-item Ascertain Dementia questionnaire [AD8]); behavioral lifestyles (smoking, drinking, sleep disorders, and physical activity); interpersonal networks (marital status, living alone, primary caregiver and social activities); living/working conditions (education level and residence type); and policy environment (pension). Univariate analyses and multivariable logistic regression were performed to identify factors independently associated with PSD.

**Results:**

Among the 482 participants, 162 (33.6%) presented with depressive symptoms, as indicated by a PHQ-9 score ≥ 10. In the multivariable analysis, factors significantly associated with PSD were identified from three ecological dimensions: personal characteristics, behavioral lifestyles, and interpersonal networks. Specifically, physical disability (mRS score > 2; OR = 4.383, 95% CI = 2.028–9.474, *p* < 0.001), cognitive impairment (AD8 score > 1; OR = 6.033, 95% CI = 3.411–10.669, *p* < 0.001), sleep disorders (OR = 6.756, 95% CI = 3.737–12.215, *p* < 0.001), and living alone (OR = 4.417, 95% CI = 1.240–15.741, *p* = 0.022) were associated with higher odds of PSD, whereas regular physical activity (> twice per week; OR = 0.064, 95% CI = 0.020–0.203, *p* < 0.001) and frequent participation in social activities (OR = 0.236, 95% CI = 0.092–0.603, *p* = 0.003) were associated with lower odds of PSD.

**Conclusion:**

PSD in older stroke patients was associated with factors across the personal, behavioral, and interpersonal dimensions of Health Ecology Theory. These findings suggest that comprehensive care may be considered to address functional and cognitive rehabilitation, sleep management, promotion of physical activity, and strengthened social support.

## 1 Introduction

Post-stroke depression (PSD) is a prevalent and serious complication after stroke, characterized by depressed mood, loss of interest, irritability, and fatigue (1). PSD affects approximately one-third of stroke survivors and represents a substantial global clinical burden (2, 3). In China, this challenge is particularly prominent because of population aging. Among older stroke patients, the reported prevalence of PSD ranged from 16.0% to 43.9% (4). PSD negatively affects functional recovery and health-related quality of life (1). Older stroke patients are especially vulnerable to PSD because of age-related functional decline and comorbidities, which may also limit antidepressant use due to concerns about adverse medication effects. Untreated PSD has been associated with increased risks of stroke recurrence, suicidal ideation, and overall mortality (4).

Given these therapeutic limitations and severe consequences, understanding factors associated with PSD is crucial. However, PSD is influenced by complex interactions among biological, psychological, behavioral, and social variables. This complexity makes reliable prediction challenging, and findings across studies have often been inconsistent (2, 3, 5, 6). Existing studies and prediction models mainly focused on acute clinical severity indices, laboratory biomarkers, or structural brain alterations, which failed to capture the broader ecological context of older patients (3, 4, 7). Recent systematic reviews also indicated that PSD prediction tools had a high risk of bias and possible overfitting (1, 7, 8). Therefore, further researches are expected to move beyond isolated clinical indicators and consider the interaction between individuals and their surrounding environmental layers to inform more individualized intervention strategies (1, 9).

Health Ecology Theory, as described by Reifsnider, Gallagher, and Forgione (2005), refers to a framework in which health outcomes are shaped by interactions between individuals and their surrounding environmental systems (10). The framework classifies health-related factors into five dimensions: personal characteristics, behavioral lifestyles, interpersonal networks, living and working conditions, and policy environment (11). This framework has been applied to identify the influencing factors of successful aging, health promotion behaviors after percutaneous coronary intervention, and sarcopenia (11–13).

Given that PSD shares similar multidimensional characteristics with these health outcomes, Health Ecology Theory may provide a suitable framework for examining PSD-related factors. However, evidence evaluating PSD through this ecological lens remains limited. A better understanding of PSD-related factors across different ecological dimensions may help identify high-risk patients earlier and support the development of targeted interventions at the individual, behavioral, and social levels. Therefore, this study aimed to identify multidimensional factors associated with PSD in older patients under the framework of Health Ecology Theory, to provide a basis for targeted and comprehensive interventions.

## 2 Materials and methods

### 2.1 Participants

This study was conducted at a tertiary hospital in Jingzhou that is designated as a national advanced stroke center and serves as the leading regional center for stroke prevention and control. Eligible patients were those hospitalized between January 1 and December 31, 2025. The study protocol was submitted to the Ethics Committee of the hospital on March 12, 2026, and was approved on March 24, 2026 (Approval No. 2026-088-01). After ethics approval, anonymized data were extracted from the hospital information system and stroke-specific follow-up records beginning in April 2026. Because this study used existing routinely collected records and involved no direct contact with patients, the requirement for informed consent was waived.

Inclusion criteria were: 1) had a first-ever stroke diagnosed according to ICD-10 criteria and confirmed by cranial CT or MRI; and 2) were aged ≥ 65 years.

Exclusion criteria were: 1) had a history of depression or other severe psychiatric disorders before stroke; or 2) had severe comorbid physical conditions, such as advanced malignancy or major organ failure.

Sample size was calculated based on Riley’s criteria for the clinical model to ensure robust model performance and minimize overfitting (14). Based on previous literature, the anticipated post-stroke depression prevalence was set at 33% (6), with an expected model discrimination (AUC) of 0.81 (5) (corresponding to a Cox-Snell R^2^ of approximately 0.253). The sample size was assessed based on eight candidate variables included in the multivariable logistic regression model. The minimum required sample size was estimated using the pmsampsize package in RStudio. The calculation indicated that at least 340 participants, including 113 outcome events, were required. The final sample of 482 participants, including 162 participants classified as having PSD, exceeded this requirement. Furthermore, this calculated sample size also satisfies the traditional rule of requiring at least 10 events per variable (10 EPV) to ensure model stability.

### 2.2 Measurement instruments

#### 2.2.1 The patient health questionnaire-9

The Patient Health Questionnaire-9 (PHQ-9), recommended by the American Heart Association/American Stroke Association (AHA/ASA), was used to assess depressive symptom severity (15). PHQ-9 is a self-reported instrument that assesses each item on a 4-point scale ranging from 0 (“not at all”) to 3 (“nearly every day”), with a total score between 0 and 27 (16). Based on the established scoring system, PHQ-9 total scores are categorized as follows: 0–4 indicates no or minimal depressive symptoms, 5–9 mild depressive symptoms, 10–14 moderate depressive symptoms, 15–19 moderately severe depressive symptoms, and 20–27 severe depressive symptoms. For analytical purposes, participants with PHQ-9 scores ≥ 10 were classified as the PSD group, a threshold originally validated in primary care settings with a sensitivity of 88% and a specificity of 88% (16).

#### 2.2.2 The modified rankin scale

The modified Rankin Scale (mRS) was used to assess post-stroke functional disability. The mRS ranges from 0 (no symptoms) to 6 (death), with higher scores indicating greater disability. The scale demonstrated strong test-retest reliability (kappa = 0.81 to 0.95) and acceptable inter-rater reliability (17). According to commonly used clinical grouping, patients with an mRS score of 0–2 were classified as functionally independent, whereas those with an mRS score > 2 were classified as having moderate to severe disability requiring assistance in daily life.

#### 2.2.3 The eight-item ascertain dementia questionnaire

Cognitive impairment was assessed using the Eight-item Ascertain Dementia questionnaire (AD8), a brief and highly sensitive instrument designed to detect early cognitive changes. A primary advantage of the AD8 is its administrative simplicity and flexibility, as it can be reliably completed by either the patient or a caregiver. A recent systematic review and meta-analysis showed that, at a cutoff score of ≥2, the AD8 demonstrated acceptable diagnostic accuracy for cognitive impairment in older adults, with pooled sensitivity and specificity of 88% and 75%, respectively (18). Each AD8 item evaluates whether the patient has shown a change compared with previous functioning; the presence of a change is scored as 1 and no change is scored as 0, with a total score ranging from 0 to 8. In this study, a total score of ≥ 2 was adopted as the clinical cutoff indicating the presence of cognitive impairment, whereas a score of 0 or 1 suggests normal cognition (19).

### 2.3 Data collection and variable definitions

Data were extracted from two routinely collected sources: the hospital information system and stroke-specific follow-up records, using a standardized data-collection form. Two researchers independently extracted the data and cross-checked the results to ensure accuracy. Relatively stable demographic and clinical variables, including sex, age, diabetes, hypertension, hyperlipidemia, coronary heart disease, acute treatment, marital status, education level, residence type, and pension status, were extracted from the hospital medical records. Variables reflecting current status at one month after discharge were extracted from the stroke-specific follow-up records, including mRS score, AD8 score, BMI, smoking, drinking, sleep disorders, physical activity, living alone, primary caregiver, and social activities.

Smoking and alcohol consumption were classified according to current use at follow-up. Self-reported sleep disorders were defined as difficulty initiating or maintaining sleep, early-morning awakening, or poor sleep quality. Physical activity included purposeful activities such as walking or exercise, while social activities included participation in community, recreational, religious, volunteer, or other organized group activities. Based on self-reported usual weekly frequency at follow-up, physical activity was categorized as ≤2 or >2 times per week, and social activity as ≤1 or >1 time per week. Living alone was defined as residing without other household members, and primary caregivers were categorized as spouses, adult children, or others. Marital status was categorized as married or unmarried, with single, divorced, and widowed participants grouped as unmarried.

### 2.4 Statistical analysis

All statistical analyses were performed using SPSS version 27.0. Continuous variables were presented using mean ± standard deviation, while categorical variables were presented as frequencies and percentages. Normally distributed continuous variables were compared between groups using independent-samples t-tests, while categorical variables were compared using chi-square tests. The level of statistical significance was set at *p* < 0.05 (two-tailed). PSD status (PHQ-9 score ≥ 10 vs. < 10) was entered as the dependent variable. Variables with *p* < 0.05 in the univariate analyses were simultaneously entered into a multivariable binary logistic regression model using the enter method. Odds ratios (ORs) and corresponding 95% confidence intervals (CIs) were reported. Model fit was assessed using the Hosmer–Lemeshow goodness-of-fit test. Model discrimination was evaluated using the area under the receiver operating characteristic curve (AUC), equivalent to the C-statistic. Multicollinearity among independent variables was assessed using tolerance and the variance inflation factor (VIF).

## 3 Results

### 3.1 Participant characteristics and univariate analysis

A total of 482 older stroke patients were included, of whom 162 (33.6%) evaluated positive for clinically significant depressive symptoms based on a PHQ-9 score ≥ 10 and were classified into the PSD group for analysis.

According to Health Ecology Theory, the candidate factors were categorized into five dimensions. As shown in **Table 1**, significant differences between the PSD and non-PSD groups were observed in mRS score (χ² = 18.620, *p* < 0.001), AD8 score (χ² = 115.424, *p* < 0.001), sleep disorders (χ² = 81.969, *p* < 0.001), physical activity (χ² = 76.077, *p* < 0.001), marital status (χ² = 11.318, *p* < 0.001), living alone (χ² = 46.038, *p* < 0.001), primary caregiver (χ² = 40.778, *p* < 0.001), and social activities (χ² = 55.998, *p* < 0.001). No statistically significant differences were observed for the other candidate factors (all *p* > 0.05).

**Table 1:** Univariate analysis of candidate factors.

Candidate Factors		Total (n = 482)	PSD (n = 162)	Non-PSD (n = 320)	t/χ²	*p*
Personal characteristics						
Sex	Male	275 (57.1)	83 (51.2)	192 (60.0)	3.373	0.066
	Female	207 (42.9)	79 (48.8)	128 (40.0)		
Age (Mean ± SD)		73.30 ± 5.44	73.54 ± 5.19	73.18 ± 5.57	0.683	0.495
mRS score	≤ 2	415 (86.1)	124 (76.5)	291 (90.9)	18.620	**<0.001**
	> 2	67 (13.9)	38 (23.5)	29 (9.1)		
AD8 score	≤ 1	332 (68.9)	60 (37.0)	272 (85.0)	115.424	**< 0.001**
	> 1	150 (31.1)	102 (63.0)	48 (15.0)		
BMI (Mean ± SD)		23.58 ± 3.40	23.47 ± 3.41	23.64 ± 3.40	0.509	0.611
Diabetes	Yes	137 (28.4)	41 (25.3)	96 (30.0)	1.164	0.281
	No	345 (71.6)	121 (74.7)	224 (70.0)		
Hypertension	Yes	341 (70.7)	115 (71.0)	226 (70.6)	0.007	0.934
	No	141 (29.3)	47 (29.0)	94 (29.4)		
Hyperlipidemia	Yes	69 (14.3)	23 (14.2)	46 (14.4)	0.003	0.958
	No	413 (85.7)	139 (85.8)	274 (85.6)		
CHD^*^	Yes	51 (10.6)	18 (11.1)	33 (10.3)	0.072	0.788
	No	431 (89.4)	144 (88.9)	287 (89.7)		
Acute Treatment	EVT^*^	37 (7.7)	10 (6.2)	27 (8.4)	2.067	0.356
	IVT^*^	35 (7.3)	15 (9.3)	20 (6.3)		
	No	410 (85.1)	137 (84.6)	273 (85.3)		
Behavioral lifestyles						
Smoking	Yes	111 (23.0)	35 (21.6)	76 (23.8)	0.279	0.597
	No	371 (77.0)	127 (78.4)	244 (76.3)		
Drinking	Yes	97 (20.1)	28 (17.3)	69 (21.6)	1.225	0.268
	No	385 (79.9)	134 (82.7)	251 (78.4)		
Sleep disorders	Yes	170 (35.3)	102 (63.0)	68 (21.3)	81.969	**< 0.001**
	No	312 (64.7)	60 (37.0)	252 (78.8)		
Physical activity	≤ twice per week	346 (71.8)	157 (96.9)	189 (59.1)	76.077	**<0.001**
	> twice per week	136 (28.2)	5 (3.1)	131 (40.9)		
Interpersonal networks						
Marital status	Married	414 (85.9)	127 (78.4)	287 (89.7)	11.318	**< 0.001**
	Unmarried	68 (14.1)	35 (21.6)	33 (10.3)		
Living alone	Yes	82 (17.0)	54 (33.3)	28 (8.8)	46.038	**< 0.001**
	No	400 (83.0)	108 (66.7)	292 (91.3)		
Primary caregiver	Spouse	146 (30.3)	28 (17.3)	118 (36.9)	40.778	**< 0.001**
	Adult children	260 (53.9)	87 (53.7)	173 (54.1)		
	Others	76 (15.8)	47 (29.0)	29 (9.1)		
Social activities	≤ once per week	357 (74.1)	154 (95.1)	203 (63.4)	55.998	**< 0.001**
	> once per week	125 (25.9)	8 (4.9)	117 (36.6)		
Living and working conditions						
Education level	≤ 12 years	381 (79.0)	136 (84.0)	245 (76.6)	3.544	0.060
	> 12 years	101 (21.0)	26 (16.0)	75 (23.4)		
Residence type	Urban	228 (47.3)	67 (41.4)	161 (50.3)	3.460	0.063
	Rural	254 (52.7)	95 (58.6)	159 (49.7)		
Policy environment						
Pension	Yes	145 (30.1)	44 (27.2)	101 (31.6)	0.991	0.320
	No	337 (69.9)	118 (72.8)	219 (68.4)		

^*^ CHD, Coronary Heart Disease; EVT, Endovascular Therapy; IVT, Intravenous Thrombolysis.

Bold values indicate *p* < 0.05.

### 3.2 Multivariable logistic regression analysis

**Figure 1:**
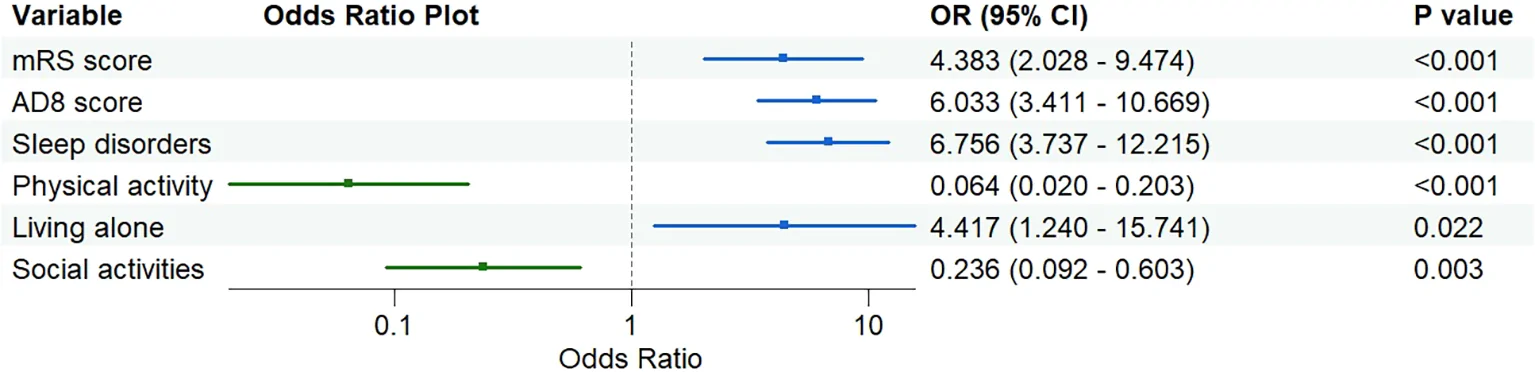
Forest plot of factors associated with PSD in the multivariable logistic regression model.

Variables with *p* < 0.05 in the univariate analyses were simultaneously entered into a multivariable binary logistic regression model using the enter method. PSD status (PHQ-9 score ≥ 10 vs. < 10) was entered as the dependent variable. As shown in **Table 2**; **Figure 1**, factors independently associated with PSD were identified across three dimensions of Health Ecology Theory: personal characteristics, behavioral lifestyles, and interpersonal networks. The Hosmer–Lemeshow goodness-of-fit test was non-significant (χ² = 10.713, df = 8, *p* = 0.219), indicating no evidence of poor model fit. The final model showed an AUC/C-statistic of 0.917 (95% CI: 0.892-0.942). No serious multicollinearity was detected, with tolerance values ranging from 0.618 to 0.929 and VIF values ranging from 1.076 to 1.619.

**Table 2:** Multivariable binary logistic regression analysis.

Variable		Code	β	SE	Wald	*p*	OR	95% CI
Personal characteristics								
mRS score	≤ 2	0						
	> 2	1	1.478	0.393	14.122	**< 0.001**	4.383	2.028-9.474
AD8 score	≤ 1	0						
	> 1	1	1.797	0.291	38.177	**< 0.001**	6.033	3.411-10.669
Behavioral lifestyles								
Sleep disorders	No	0						
	Yes	1	1.910	0.302	39.975	**< 0.001**	6.756	3.737-12.215
Physical activity	≤ twice per week	0						
	> twice per week	1	-2.746	0.588	21.834	**< 0.001**	0.064	0.020-0.203
Interpersonal networks								
Marital status	Unmarried	0						
	Married	1	-0.578	0.393	2.168	0.141	0.561	0.260-1.211
Living alone	No	0						
	Yes	1	1.486	0.648	5.250	**0.022**	4.417	1.240-15.741
Primary caregiver	Others	0						
	Adult children	1	-0.310	0.651	0.227	0.634	0.733	0.205-2.628
	Spouse	2	-0.381	0.697	0.299	0.585	0.683	0.174-2.677
Social activities	≤ once per week	0						
	> once per week	1	-1.445	0.479	9.089	**0.003**	0.236	0.092-0.603

Multivariable binary logistic regression using the enter method. Bold values indicate p < 0.05.

Within the personal characteristics dimension, mRS score > 2 (OR = 4.383, 95% CI = 2.028–9.474, *p* < 0.001) and AD8 score > 1 (OR = 6.033, 95% CI = 3.411–10.669, *p* < 0.001) were associated with higher odds of PSD. Within the behavioral lifestyles dimension, sleep disorders were associated with higher odds of PSD (OR = 6.756, 95% CI = 3.737–12.215, *p* < 0.001). In contrast, engaging in physical activity more than twice per week was associated with lower odds of PSD (OR = 0.064, 95% CI = 0.020–0.203, *p* < 0.001). Within the interpersonal networks dimension, living alone was associated with higher odds of PSD (OR = 4.417, 95% CI = 1.240–15.741, *p* = 0.022), whereas participating in social activities more than once per week was associated with lower odds of PSD (OR = 0.236, 95% CI = 0.092–0.603, *p* = 0.003). Marital status and primary caregiver were not independently associated with PSD in the multivariable model (all *p* > 0.05).

## 4 Discussion

Guided by Health Ecology Theory, this study examined multidimensional factors associated with PSD among older stroke patients. The results showed 33.6% of older stroke participants were complicated with depressive symptom. This frequency was consistent with the general estimate (3, 9, 15). Significant associations were observed across three ecological dimensions: personal characteristics, behavioral lifestyles, and interpersonal networks. These findings suggest that PSD is associated with a combination of neurobiological, behavioral, and psychosocial factors.

In the personal characteristics dimension, greater functional disability and cognitive impairment were associated with higher odds of PSD. Patients with an mRS score > 2 had approximately four times the odds of PSD, while those with an AD8 score > 1 had approximately six times the odds. These findings were consistent with previous evidence showing that functional dependence, stroke-related disability, and cognitive impairment are closely associated with depressive symptoms after stroke (1, 9, 15, 20). Studies found that functional disability may reduce independence, limit patients’ ability to perform family and social roles, and weaken perceived self-efficacy (21). Cognitive impairment may further affect emotional regulation, stress processing, treatment participation, and coping ability. Within the framework of Health Ecology Theory, these personal characteristics may interact with the surrounding environment and contribute to psychological distress. Therefore, early functional and cognitive assessments may help identify patients who need closer psychological monitoring, and individualized rehabilitation, psychological support, and appropriate cognitive interventions may be considered as components of comprehensive care.

In the behavioral lifestyles dimension, sleep disorders were associated with higher odds of PSD, whereas regular physical activity was associated with lower odds. This finding highlighted the role of modifiable daily behaviors in the psychological adjustment of older stroke patients. Sleep disturbances may coexist with fatigue, emotional dysregulation, and reduced resilience during stroke recovery, and may either contribute to depressive symptoms or appear as part of depression itself (3, 4). In contrast, regular physical activity may support functional recovery, social engagement, self-efficacy, and psychological adaptation after stroke. However, because of the cross-sectional design, the direction of these associations cannot be determined. From a clinical practice perspective, structured sleep management and early rehabilitation activities may be practical components of post-discharge care, but longitudinal and intervention studies are needed to determine their effects.

In the interpersonal networks dimension, living alone was associated with higher odds of PSD, whereas frequent social participation was associated with lower odds. These results were highly consistent with the AHA/ASA scientific statement and recent overviews, which recognized social isolation and reduced social participation as both factors associated with depressive symptoms after stroke (4, 15). These findings emphasize the importance of social support and social engagement in post-stroke psychological adjustment. The observed associations may be explained by the stress-buffering hypothesis (22). Patients who live alone may have less immediate emotional and practical support during recovery, which may increase feelings of loneliness, helplessness, and uncertainty. Emotional support can help patients cope with fear and loss of independence, while practical support may assist with daily activities and treatment adherence. Frequent social participation may also strengthen a sense of belonging, promote communication, and help patients rebuild social roles. Within Health Ecology Theory, interpersonal connections may represent an important external resource for psychological adaptation. Family-supported care, peer support, discharge planning, and structured social participation programs may therefore be considered to strengthen interpersonal connections among older stroke patients. However, prospective studies are needed to determine whether these interventions can improve PSD-related outcomes.

In contrast, diabetes, hypertension, hyperlipidemia, and variables in the living/working conditions and policy environment dimensions were not significantly associated with PSD in this study. Although previous studies suggested that vascular burden (23, 24) and socioeconomic disadvantage (25) may be related to PSD, including hypertension, lipid-related indicators, education level, and socioeconomic status, these associations have not been fully consistent (15). In this study, diabetes, hypertension, hyperlipidemia, education level, residence type, and pension status showed limited differences. In addition, these variables were measured as broad categorical indicators and may not capture disease severity, control status, income level, economic security, or actual social resources. Therefore, more proximal post-discharge factors, such as functional disability, cognitive impairment, sleep disorders, physical activity, living alone, and social participation, may have shown stronger associations with PSD at one month after discharge.

## 5 Conclusion

Clinically significant depressive symptoms were prevalent among older stroke patients one month after discharge. Factors associated with PSD were mainly identified in the personal characteristics, behavioral lifestyles, and interpersonal networks dimensions of Health Ecology Theory. Greater functional disability, cognitive impairment, sleep disorders, and living alone were associated with higher odds of PSD, whereas regular physical activity and frequent social participation were associated with lower odds. These findings suggest that early functional and cognitive assessment, sleep management, individualized physical activity, and strengthened family and social support may be valuable components of comprehensive care for older stroke patients.

## 6 Limitations

This study has several limitations. First, because this was a cross-sectional study, the temporal sequence and causal relationships between the identified factors and PSD could not be determined. Second, this study was conducted at a single tertiary hospital, which may limit the generalizability of the findings to other settings or populations. Third, PSD was identified using the PHQ-9 rather than a structured psychiatric interview; therefore, the findings should be interpreted as factors associated with study-defined PSD or clinically significant post-stroke depressive symptoms. Fourth, sleep disorders, physical activity, and social participation were assessed using routinely collected single-item follow-up questions rather than validated standardized instruments, which may have introduced measurement bias. Finally, the AD8 was used as a brief cognitive screening tool because the one-month post-discharge follow-up was conducted by telephone or online follow-up, making it difficult to administer more comprehensive cognitive assessments such as the Mini-Mental State Examination (MMSE) or Montreal Cognitive Assessment (MoCA). However, the AD8 is primarily a dementia and cognitive impairment screening tool and has not been specifically validated for post-stroke cognitive impairment in this study population. Future multicenter longitudinal studies using structured diagnostic interviews, validated standardized instruments, and comprehensive cognitive assessments are needed to confirm these findings and clarify the temporal relationships between these factors and PSD.

## Data Availability

The raw data supporting the conclusions of this article will be made available by the authors, without undue reservation.
